# Platelet–lymphocyte ratio is a prognostic marker in small cell lung cancer—A systemic review and meta-analysis

**DOI:** 10.3389/fonc.2022.1086742

**Published:** 2023-01-13

**Authors:** Hongbin Zhou, Jiuke Li, Yiting Zhang, Zhewen Chen, Ying Chen, Sa Ye

**Affiliations:** ^1^ Cancer Center, Department of Pulmonary and Critical Care Medicine, Zhejiang Provincial People’s Hospital (Affiliated People’s Hospital, Hangzhou Medical College), Hangzhou, Zhejiang, China; ^2^ Department of Ophthalmology, Hangzhou Aier Eye Hospital, Hangzhou, Zhejiang, China; ^3^ Department of Pulmonary and Critical Care Medicine, Xianju People’s Hospital, Taizhou, Zhejiang, China; ^4^ Center for General Practice Medicine, Department of Clinical Nutrition, Zhejiang Provincial People’s Hospital (Affiliated People’s Hospital, Hangzhou Medical College), Hangzhou, Zhejiang, China

**Keywords:** small cell lung cancer (SCLC), platelet–lymphocyte ratio (PLR), overall survival (OS), progression-free survival (PFS), prognosis

## Abstract

**Aim:**

The aim of this study was to evaluate the relationship between platelet–lymphocyte ratio (PLR) and prognosis in small cell lung cancer (SCLC) patients.

**Method:**

A comprehensive search was carried out to collect related studies. Two independent investigators extracted the data of hazard ratio (HR) and 95% confidence interval (CI) for overall survival (OS) or progression-free survival (PFS). A random-effect model was applied to analyze the effect of different PLR levels on OS and PFS in SCLC patients. Moreover, subgroup analysis was conducted to seek out the source of heterogeneity.

**Results:**

A total of 26 articles containing 5,592 SCLC patients were included for this meta-analysis. SCLC patients with a high PLR level had a shorter OS compared with patients with a low PLR level, in both univariate (HR = 1.56, 95% CI 1.28–1.90, *p* < 0.0001) and multivariate (HR = 1.31, 95% CI 1.08–1.59, *p* = 0.007) models. SCLC patients with a high PLR level had a shorter PFS compared with patients with a low PLR level, in the univariate model (HR = 1.71, 95% CI 1.35–2.16, *p* < 0.0001), but not in the multivariate model (HR = 1.17, 95% CI 0.95–1.45, *p* = 0.14). Subgroup analysis showed that a high level of PLR shortened OS in some subgroups, including the Asian subgroup, the younger subgroup, the mixed-stage subgroup, the chemotherapy-dominant subgroup, the high-cutoff-point subgroup, and the retrospective subgroup. PLR level did not affect OS in other subgroups.

**Conclusion:**

PLR was a good predictor for prognosis of SCLC patients, especially in patients received chemotherapy dominant treatments and predicting OS.

**Systematic review registration:**

https://www.crd.york.ac.uk/PROSPERO/, identifier CRD42022383069.

## Introduction

Lung cancer is one of the most common tumors in the world. According to Global Cancer Statistics, approximately 2.2 million patients were diagnosed with lung cancer and 1.8 million patients died from this disease in 2020 (1). Generally, lung cancer is divided into two types, non-small cell lung cancer (NSCLC) and small cell lung cancer (SCLC). In the past years, the prognosis of NSCLC has been improved, accompanied by the wide application of target therapy and immunotherapy. Comparatively, the prognosis of SCLC remains at a poor level, with a 5-year survival rate of no more than 10%, even if the patients receive positive therapy (2). The poor prognosis of SCLC is usually attributed to its unique biological behaviors. However, host factors, such as genetic background, occupational exposure, and inflammation level, have also been considered to be related to prognosis for SCLC patients (3, 4).

Chronic inflammatory reaction is not only the initiating factor of various kinds of cancer, but also a predictor of curative effect and survival (5). Many inflammation indexes have been applied to evaluate the prognosis of lung cancer patients, and platelet–lymphocyte ratio (PLR) is such an index that appeared frequently in relevant studies. The majority of these studies were about NSCLC, and the results of the remaining studies concerning SCLC were not consistent (6–31). In most studies, a high level of PLR indicated poor prognosis (7, 8, 10, 13, 16, 19, 24–27, 30, 31). However, in several studies, the levels of PLR were not associated with prognosis of SCLC patients (9, 11, 12, 22, 23, 28). On the contrary, another study presented the result that the patients with high PLR had a better prognosis than those with low PLR (21). The great difference among these studies might be due to different populations, sample sizes, stages of disease, and treatment regimens. Thus, we conducted this meta-analysis in order to clarify the relationship between PLR and prognosis of patients with SCLC.

## Methods

### Search strategy

A comprehensive strategy was adopted to search appropriate studies about PLR and SCLC. We searched relevant articles in several databases, including PubMed, Embase, OVID, and CNKI. The terms we used were as follows: “small cell lung cancer”, “SCLC”, “platelet–lymphocyte ratio”, “PLR”, “prognosis”,” overall survival”, “OS”, “progression-free survival”, and “PFS”. In addition, other studies that met our standard were identified by manual search from references of reviews or original articles on this topic. The last entrance of these databases was on 21 October 2022. The detailed information of the search strategy is listed in the **Supplementary Material**.

### Study selection

The studies selected for further analysis should meet the following criteria (1): The subjects with SCLC were divided into two groups according to PLR value, and a precise cutoff point of PLR was given in the study (2); the endpoints of the study included overall survival (OS) and/or progression-free survival (PFS) (3); hazard ratio (HR) and 95% confidence interval (CI) for OS or PFS should be provided or could be calculated by other data in the study. The studies that did not provide sufficient data were excluded. The studies containing NSCLC or other cancer types, in which the data of SCLC could not be obtained alone, would be excluded as well.

### Data extraction

The data were independently extracted from all eligible publications by two authors according to the inclusion criteria listed above. Any disagreements were resolved by discussions with a third person. The information extracted from the studies included author, publication year, nationality, sample size, age, smoking status, stage, therapy, cutoff point of PLR, study design, HR, and 95% CI.

### Quality assessment

All the included studies were assessed with the help of the Newcastle–Ottawa Scale (NOS), which was mainly about three aspects composed of selection, comparability, and exposure (32). Each study was assigned a score from 0 to 9 points, and higher points indicated higher quality.

### Statistical analysis

HR and 95% CIs were used to assess the relative risk of OS and PFS between SCLC patients with low PLR and SCLC patients with high PLR in the studies.

The *I* ^2^ statistic was used to quantify the degree of heterogeneity, with *I* ^2^ < 25%, 25%–75%, and >75% representing low, moderate, and high degrees of inconsistency, respectively (33, 34). In the analysis of pooled data, we used two different models according to the traits of the included studies: If no heterogeneity was found, a fixed-effect model was adopted or a random-effect model was applied. If heterogeneity existed across studies, a subgroup analysis was performed to seek out the source of heterogeneity. The studies were subdivided by ethnicity (Asian dominant vs. white dominant), age (older vs. younger vs. unknown), stage (limited stage vs. extensive stage vs. mixed stage), treatment (surgery dominant vs. chemotherapy dominant vs. other therapy), study design (prospective vs. retrospective), and cutoff point (≥150 vs. <150). Sensitivity analysis was also performed to check the stability of the overall effect.

We made use of a Begg’s funnel plot to examine the underlying publication bias and Egger’s weighted regression method to calculate a *p*-value for bias (35, 36). If no publication bias exists, the funnel plot appears symmetrical.

All analyses were conducted with the use of Review Manager, V.5.2 (Revman, The Cochrane Collaboration, London, UK) or STATA software, V.12.0 (StataCorp LP, College Station, TX, USA).

## Results

### Characteristics of included studies

**Figure 1:**
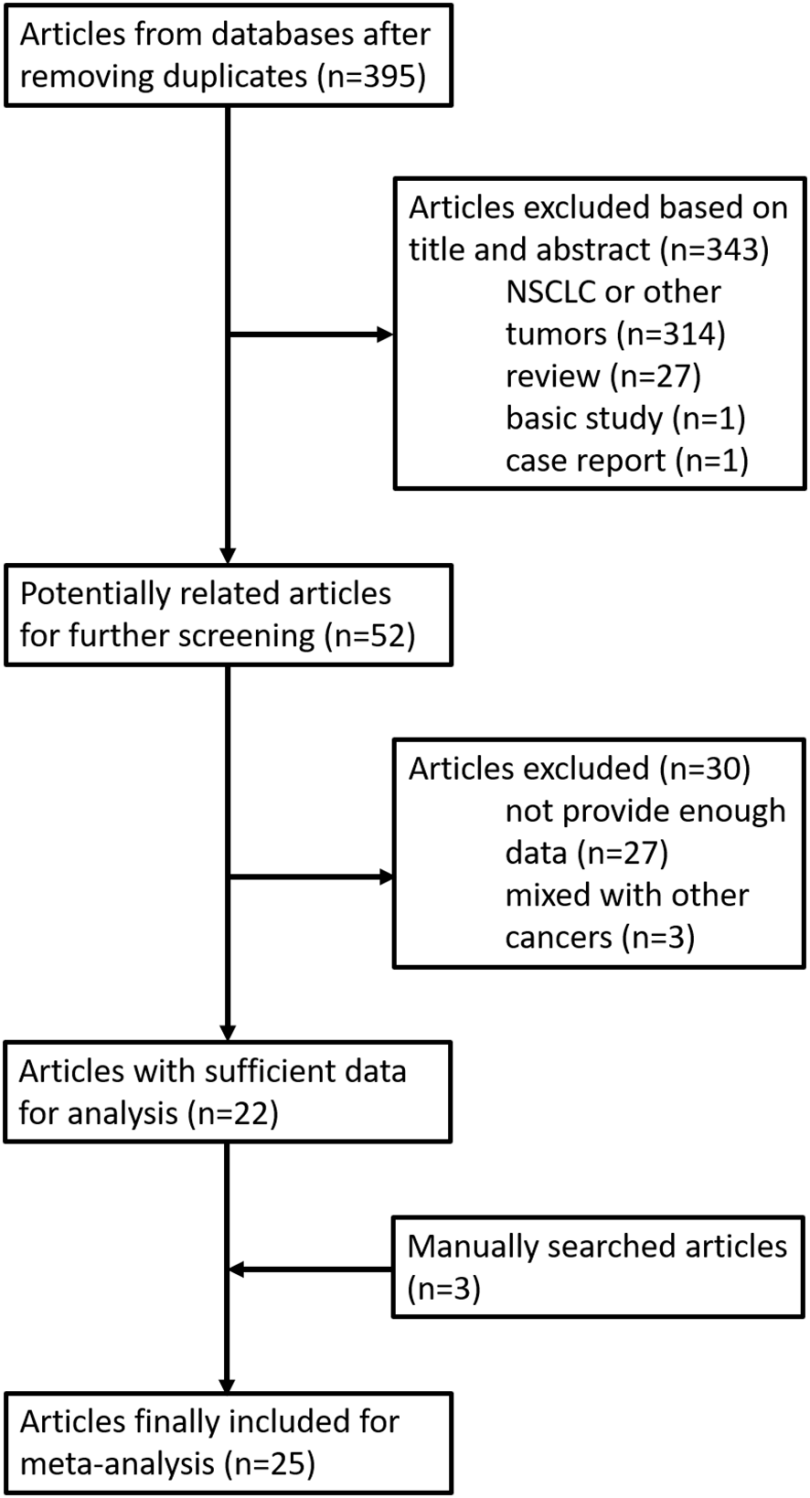
The flowchart of the study selection process for the meta-analysis.

We searched 395 potentially related articles, of which 343 articles were excluded. Most of these studies (314 articles) were about NSCLC or other tumors rather than SCLC. Moreover, 27 reviews, one basic study, and one case report were also eliminated. The remaining 52 studies have undergone further screening, and 29 studies were also excluded due to lack of necessary data. In addition, three articles were adopted by manual search from references of reviews. Finally, a total of 26 articles containing 5,592 SCLC patients were included for this meta-analysis (**Figure 1**). There were 24 studies presenting HR and 95% CI data for OS and 10 studies providing HR and 95% CI data for PFS. The characteristics of included studies are listed in **Table 1**. All patients with SCLC were diagnosed by pathology. The treatment regimens included surgery, chemotherapy, radiotherapy, and immunotherapy. The scores of included studies ranged from 5 to 9 by NOS.

**Table 1:** Characteristics of included studies.

Author	Year	Country	Sample size	Sex (M/F)	Age	Smoking status (Y/N)	Stage	Treatment	PLR cutoff point	Outcome		Design	NOS
										OS	PFS		
Liu	2017	China	139	107/32	58.4 ± 10.5	100/39	LS+ES	Surgery/Chemotherapy/Radiotherapy	148	Uni	–	Retrospective	7
Li	2018	China	58	49/9	58.98 ± 8.47	40/18	LS+ES	Surgery and/or Chemoradiation	101.8	Uni	–	Retrospective	8
Lohinai	2019	Hungary, Italy, Russia	155	114/41	58	141/14	LS	Surgery	111.253	Uni	Uni	Retrospective	8
Shen	2019	China	138	108/30	60.96 ± 8.70	NA	LS+ES	Chemotherapy ± Radiotherapy	190	–	Uni + Multi	Retrospective	6
Wu	2020	China	146	114/32	57 (19–74) *	108/38	LS+ES	Chemotherapy ± Radiotherapy	165	Uni + Multi	Uni + Multi	Retrospective	9
Chen	2021	China	299	255/44	59.4 ± 8.6	239/60	LS	Surgery	156.7	Uni + Multi	–	Retrospective	8
Yuan	2021	China	71	60/11	62 (30–77) *	71/0	ES	Radiation + Chemotherapy	210	Uni + Multi	–	Retrospective	7
Sakin	2019	Turkey	113	92/21	61 (35–83) *	113/0	ES	Chemotherapy	150	Uni	–	Retrospective	8
Wang DY	2019	China	228	159/69	58 (39–71) *	181/47	LS+ES	Chemoradiotherapy	125	Uni + Multi	Uni + Multi	Prospective	9
Suzuki	2019	USA	122	61/61	65 (60–72) *	118/4	LS	Chemoradiotherapy	140.1	Uni + Multi	–	Retrospective	8
Zhang	2019	China	286	202/84	57.7± 25.33	161/125	LS	Surgery ± Chemoradiotherapy	152.1	Multi	Multi	Retrospective	8
Suzuki	2018	USA	252	119/133	63 (56–69) *	247/5	ES	Chemoradiotherapy	194.7	Uni	–	Retrospective	7
Xie a	2015	USA	555	318/237	66.7 ± 10.3	543/12	ES	Chemotherapy ± Radiotherapy	210	Multi	–	Prospective	9
Xie b	2015	USA	383	182/201	66.7 ± 10.0	378/5	LS	Chemotherapy ± Radiotherapy	210	Multi	–	Prospective	9
Kang	2014	Korea	187	162/25	68 (43–84)*	172/15	LS + ES	Chemotherapy ± Radiotherapy	160	Multi	Multi	Retrospective	8
Huang	2021	China	358	286/72	60 (53–66)*	276/82	ES	Chemotherapy ± Radiotherapy	76	Uni + Multi	Uni	Retrospective	8
Qi	2021	China	53	34/19	NA	NA	ES	Chemotherapy + Immunotherapy	119.23	Uni + Multi	–	Prospective	5
Xiong	2021	China	41	36/5	61 (42–80)*	35/6	LS + ES	Immunotherapy	169		Uni	Retrospective	6
Pan	2017	China	275	239/36	62.59 ± 9.29	193/82	LS+ES	Chemotherapy/Surgery + Radiotherapy	258	Uni + Multi	–	Retrospective	8
Hong	2015	China	919	635/284	56 (16–84) *	567/352	LS + ES	Chemotherapy ± Radiotherapy	250	Multi	–	Retrospective	8
Rice	2021	USA	51	26/25	64.6 ± 9.0	NA	ES	Chemotherapy/Immunotherapy	320	Uni	Uni	Retrospective	7
Sonehara	2019	Japan	83	70/13	72 (43–86)*	79/4	ES	Chemotherapy	200	Uni + Multi	–	Retrospective	7
Xu	2020	China	44	41/3	NA	34/10	ES	Chemotherapy/Radiotherapy	142	Uni + Multi	–	Prospective	6
Wang L	2017	China	172	113/59	55.78 ± 10.98	144/28	LS + ES	Surgery/Chemotherapy/Radiotherapy	191	Multi	–	Retrospective	7
Wang LW	2019	China	165	125/40	58 (24–78) *	107/58	LS + ES	Chemotherapy ± Radiotherapy	189	Multi	Multi	Retrospective	8
Mao	2021	China	118	89/29	63 (35–84) *	NA	LS + ES	Chemotherapy	150	Uni	–	Prospective	6
Wang X	2017	China	181	147/34	NA	139/42	LS + ES	Chemotherapy ± Radiotherapy	178	Uni + Multi	–	Retrospective	8

PLR, platelet–lymphocyte ratio; OS, overall survival; PFS, progression-free survival; NOS, Newcastle–Ottawa Scale; NA, not available; LS, limited stage; ES, extensive stage; Uni, univariate model; Multi, multivariate model.

* The data of age were shown as median (range).

### The effect of PLR on overall survival

There were 24 studies evaluating the effect of different PLR levels on OS for SCLC patients. Among them, PLR was considered as univariable in 18 studies, while it was analyzed in multivariable models in 17 studies. As a result, SCLC patients with a high PLR level had a shorter OS compared with patients with a low PLR level, in both univariate (HR = 1.56, 95% CI 1.28–1.90, *p* < 0.0001) and multivariate (HR = 1.31, 95% CI 1.08–1.59, *p* = 0.007) models.

**Figure 2:**
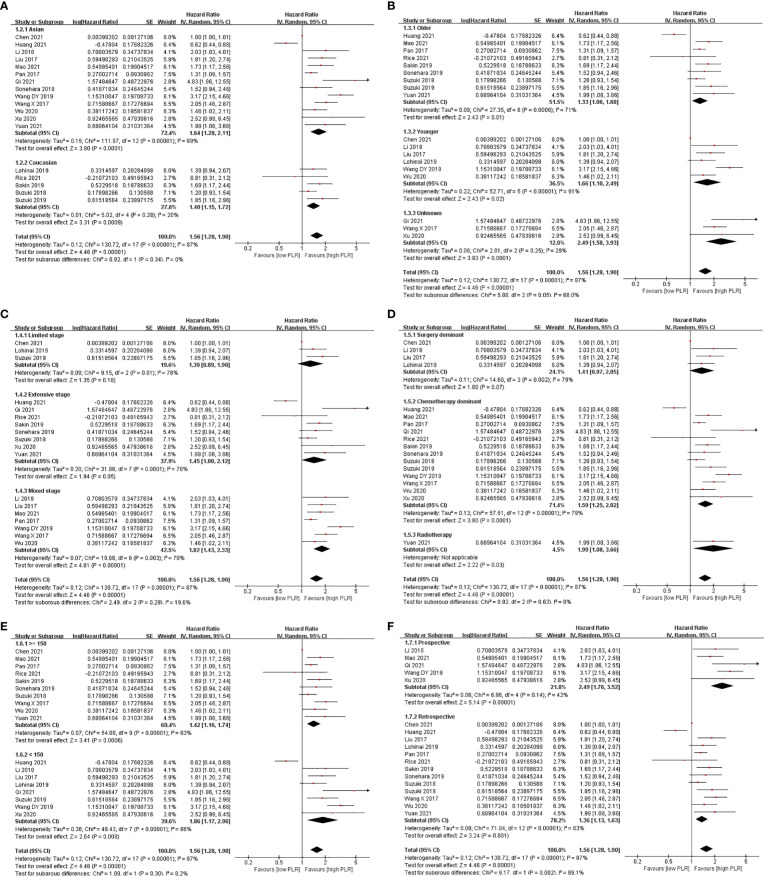
Subgroup analysis of associations between PLR and OS in SCLC patients summarized by univariate model. **(A)** Ethnicity; **(B)** age; **(C)** stage; **(D)** treatment; **(E)** cutoff point; **(F)** design.

**Figure 3:**
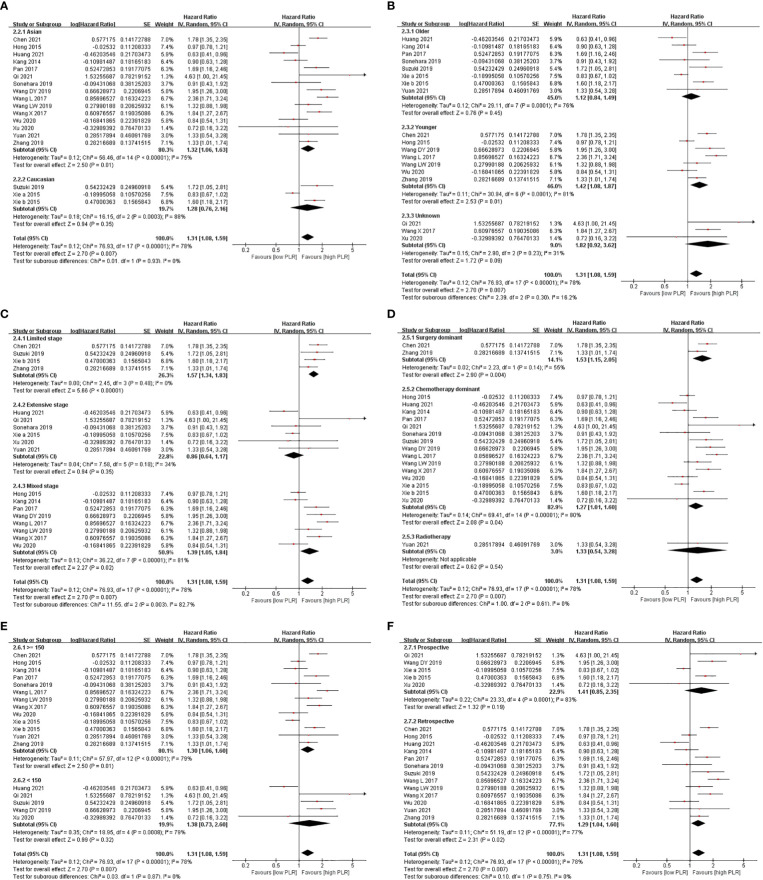
Subgroup analysis of associations between PLR and OS in SCLC patients summarized by multivariate model. **(A)** Ethnicity; **(B)** age; **(C)** stage; **(D)** treatment; **(E)** cutoff point; **(F)** design.

Subgroup analysis showed that a high level of PLR shortened OS in majority of the subgroups, except the limited-stage subgroup, the extensive-stage subgroup, and the surgery-dominant subgroup, when PLR was analyzed in the univariate model. While PLR was analyzed in the multivariate model, the result that a high level of PLR shortened OS appeared in fewer subgroups, including the Asian subgroup, the younger subgroup, the limited-stage subgroup, the mixed-stage subgroup, the surgery-dominant subgroup, the chemotherapy-dominant subgroup, the high-cutoff-point subgroup, and the retrospective subgroup (**Figures 2**, **3** and **Table 2**).

**Table 2:** Subgroup analysis about the effect of PLR level on OS for SCLC patients.

	Univariate Model				Multivariate Model			
	Studies	HR [95% CI]	*p*	*I* ^2^	Studies	HR [95% CI]	*p*	*I* ^2^
**Ethnicit**y								
Asian	13	1.64 [1.28, 2.11]	<0.0001	89%	15	1.32 [1.06, 1.63]	0.01	75%
White	5	1.40 [1.15, 1.72]	0.0009	20%	3	1.28 [0.76, 2.16]	0.35	88%
**Age**								
Older	9	1.33 [1.06, 1.68]	0.01	71%	8	1.12 [0.84, 1.49]	0.45	76%
Younger	6	1.66 [1.10, 2.49]	0.02	91%	7	1.42 [1.08, 1.87]	0.01	81%
Unknown	3	2.49 [1.58, 3.93]	<0.0001	29%	3	1.82 [0.92, 3.62]	0.09	31%
**Stage**								
Limited stage	3	1.30 [0.89, 1.90]	0.18	78%	4	1.57 [1.34, 1.83]	<0.00001	0
Extensive stage	8	1.45 [1.00, 2.12]	0.05	78%	6	0.86 [0.64, 1.17]	0.35	34%
Mixed stage	7	1.82 [1.43, 2.33]	<0.00001	70%	8	1.39 [1.05, 1.84]	0.02	81%
**Treatment**								
Surgery dominant	4	1.41 [0.97, 2.05]	0.07	79%	2	1.53 [1.15, 2.05]	0.004	55%
Chemotherapy dominant	13	1.59 [1.25, 2.02]	0.0001	79%	15	1.27 [1.01, 1.60]	0.04	80%
Radiotherapy	1	1.99 [1.08, 3.66]	0.03	NA	1	1.33 [0.54, 3.28]	0.54	NA
**Cutoff point**								
≥150	10	1.42 [1.16, 1.74]	0.0006	83%	13	1.30 [1.06, 1.60]	0.01	79%
<150	8	1.86 [1.17, 2.96]	0.008	86%	5	1.38 [0.73, 2.60]	0.32	79%
**Design**								
Prospective	5	2.49 [1.76, 3.52]	<0.00001	42%	5	1.41 [0.85, 2.35]	0.19	83%
Retrospective	13	1.36 [1.13, 1.63]	0.001	83%	13	1.29 [1.04, 1.60]	0.02	77%

### The effect of PLR on progression-free survival

**Figure 4:**
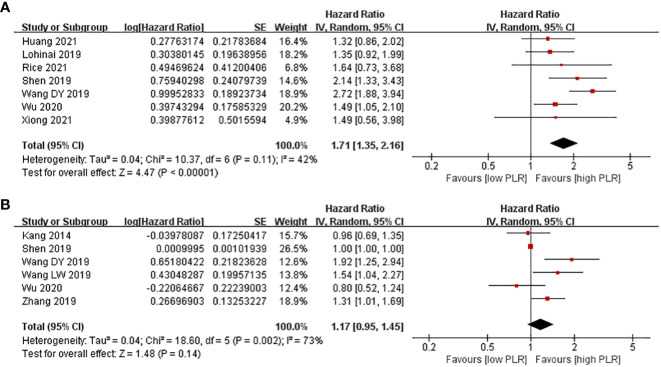
Associations between PLR and PFS in SCLC patients. **(A)** Univariate model; **(B)** multivariate model.

There were 10 studies evaluating the effect of different PLR levels on OS for SCLC patients. Among them, PLR was considered as univariable in seven studies, while it was analyzed in multivariable models in six studies. As a result, SCLC patients with a high PLR level had shorter PFS compared with patients with a low PLR level in the univariate model (HR = 1.71, 95% CI 1.35–2.16, *p* < 0.0001). However, no evident difference in PFS was observed between patients with a high PLR level and those with a low PLR level (HR = 1.17, 95% CI 0.95–1.45, *p* = 0.14) in the multivariate model (**Figure 4**).

### Sensitivity analysis

Sensitivity analysis was performed to observe whether a single study would affect the overall results. No study was found to affect the pooled HR value for OS or PFS in both univariate and multivariate models (**Figure S1**).

### Publication bias

**Figure 5:**
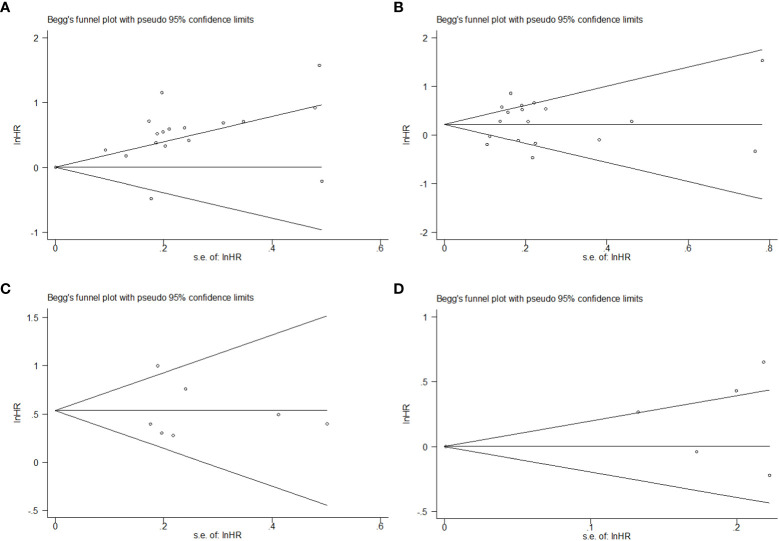
Begg’s funnel plot of HR on prognosis in SCLC patients with different levels of PLR. **(A)** HR on OS in the univariate model; **(B)** HR on OS in the multivariate model; **(C)** HR on PFS in the univariate model; **(D)** HR on PFS in the multivariate model.

Publication bias was tested using Begg’s and Egger’s tests. These tests did not show significant results in the comparison of HR for PFS. However, Egger’s test showed significant results in the comparison of HR for OS in the univariate model, but not in the multivariate model (**Table S1**). The distribution of data points revealed asymmetry, which indicated the possibility of publication bias (**Figure 5**).

## Discussion

The precise information of cancer pathogenesis largely remains unknown. However, it has been widely accepted that chronic inflammation is related to many types of cancers, including lung cancer (37–39). To measure the strength of inflammation, researchers put forward a series of indexes, such as neutrophil–lymphocyte ratio (NLR), PLR, systemic inflammation index (SII), and systemic inflammation response index (SIRI). These indexes usually appeared in various kinds of prognostic models on lung cancer as well as other cancers. Among them, NLR was most frequently mentioned and considered as a good predictor of prognosis for NSCLC patients. So far, the relationship between NLR and OS or PFS of NSCLC patients has been summarized in several meta-analyses (40–42). Comparatively, much fewer literatures were about PLR in prognosis of SCLC patients. In the present study, we collected 26 reports about the effect of PLR level on OS and/or PFS in SCLC patients. As a result, we found that high PLR led to a shorter OS and PFS as a whole, which was quite different from a previous meta-analysis by Winther-Larsen et al. (43). In that study, the authors incorporated seven original articles and revealed that PLR had no significant value for predicting OS in SCLC patients. Moreover, it was not even mentioned whether PLR contributed to predicting PFS in that study. A smaller sample size and different inclusion criteria might be the main cause of our discrepancy.

In our work, we pooled HR value according to different statistical models stated in each original study. For univariate analysis, PLR was considered as the only factor that affected OS or PFS. In fact, many factors might play a role in OS or PFS at the same time. Thus, multivariate analysis was applied to accurately evaluate the effect of a specific factor. We found that the effect of PLR on OS in the univariate model was more evident than that in the multivariate model, but the overall effects were consistent in these two models. As for the effect of PLR on PFS, it was observed that high PLR led to worse PFS only in the univariate model but not in the multivariate model. There were several reasons that might be used to explain this phenomenon. First, only seven and six articles reported the relationship between PLR and PFS in two models, respectively. The small sample size might affect the overall effect. Second, different factors were adopted to evaluate their effects on PFS in different studies. For example, Shen et al. added seven variants, including gender, age, stage, therapy, and PLR, in their model, and drew a conclusion that PLR was not associated with PFS (17). On the contrary, Wang et al. established a model with only four factors and found that a high level of PLR correlated with short PFS (31). Although PLR, stage, and therapy were adopted in both models, the remaining variants were quite different. It could not be ruled out that the effect of PLR changed a lot in different models. Third, not all the authors conducted both univariate analysis and multivariate analysis, which meant different subjects were enrolled in each model. Hence, it was not strange that the overall effect of PLR on PFS was evident in one model but not in the other.

It should be noted that great heterogeneity existed among included studies. Subgroup analysis was conducted to seek out the origin of heterogeneity. Although high PLR led to worse OS in general, the effect disappeared in the extensive-stage subgroup, with the use of both models. Compared with the patients in the limited stage, the patients in the extensive stage had a much shorter survival. It was difficult to further discriminate different survival by PLR level in SCLC patients in the extensive stage. In fact, lymphocyte or platelet number could be easily affected as a result of alteration of internal and external environment for everyone (44). On one hand, tumor-associated inflammation might stimulate the production of platelet and lymphocyte. On the other hand, hematopoietic and immunological function could be greatly inhibited as the tumor progressed. PLR level was affected by multiple factors in such complex status, which restricted it as a prognostic index for patients with SCLC in the extensive stage. For some subgroups divided by ethics, age, and treatment, the effect of PLR existed in one subgroup, but disappeared in another subgroup. These results indicated that many factors, other than PLR, might greatly affect prognosis for SCLC patients. However, in spite of potential confounding factors, our result was rather robust as evidenced by sensitivity analysis.

The traditional therapy for SCLC consists of chemotherapy, radiotherapy, and surgery if possible. In most of the included studies, chemotherapy was the dominant treatment approach, and chemotherapy plus radiotherapy was the common combination. We found that a high PLR level was associated with short OS in SCLC patients who received chemotherapy-dominant therapy in two models, which indicated that PLR was an excellent prognostic factor for these patients. We did not obtain consistent result about the association between PLR and OS in SCLC patients who received surgery in different models. The inconsistent results might be attributed to limited studies. In fact, most SCLC patients had no chance of surgery because they were already at the extensive stage once they were diagnosed. Thus, it was not surprising that only a few studies were adopted in our analysis. Nowadays, increasing evidence suggested that immunotherapy was a promising approach for SCLC treatment. However, very few studies discussed the role of PLR level in the OS and/or PFS in SCLC patients who received immunotherapy. As a novel approach, it is necessary to evaluate the effect of PLR level on prognosis of SCLC patients.

Our study had some limitations. First, majority of the studies included for analysis were from Asian countries, particularly from China. The remaining were about whites, and data regarding Africans were almost absent. The incomprehensive data might affect the reliability of this study. Second, the number of lung cancer patients in the included studies was small. The small sample size might affect the stability of the results. Third, as mentioned above, stage, treatment, and other factors might also affect the prognosis of patients. The existence of these factors might lead to confusing results. Fourth, the PLR level fluctuates and is affected by many other diseases, which can coexist with lung cancer in some patients. However, the majority of the studies did not provide information on whether lung cancer patients had concomitant diseases. Concomitant disease may be a confounding factor that affected the final results. Last, patients received more than one treatment in a large part of reports. Considering the impossibility of isolating individual patients by different treatment patterns, subdividing the studies into more than one “dominant” treatment may limit the current analysis.

## Conclusion

In conclusion, we first demonstrated that PLR was a good predictor for prognosis of SCLC patients, especially in patients received chemotherapy dominant treatments and predicting OS. Due to the various shortcomings in the present study, future studies with a larger sample size, covering different ethnicities and unifying stage and treatment, should be carried out to further validate the relationship between PLR and prognosis of this disease.

## Data availability statement

The original contributions presented in the study are included in the article/**Supplementary Material**. Further inquiries can be directed to the corresponding author.

## Author contributions

HZ, JL, and SY designed this study. YZ searched related studies, while YC and ZC extracted the data of enrolled studies. HZ was responsible for statistical analysis. JL and SY performed graph drafting. This manuscript was originally written and finally approved by all the authors.
