# Supplementary material for: Platelet–lymphocyte ratio is a prognostic marker in small cell lung cancer—A systemic review and meta-analysis
Source: Front Oncol. 2023 Jan 13;12:1086742. doi: 10.3389/fonc.2022.1086742 (PMC9880219; doi:10.3389/fonc.2022.1086742)
Supplement: Supplementary file 1 [file DataSheet_1.pdf]

Supplementary material

Sensitivity analysis of the association between PLR level and OS or PFS in SCLC patients.

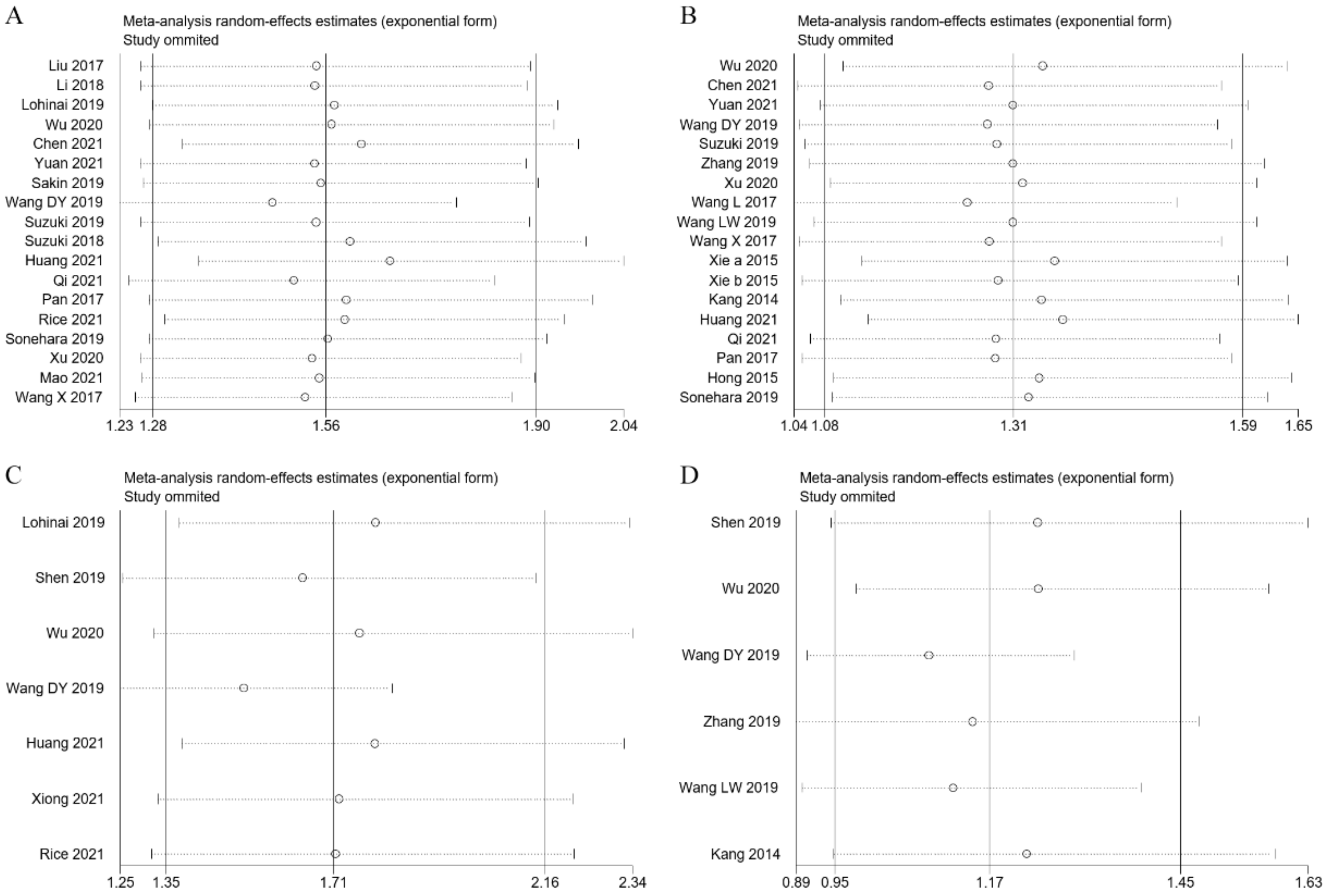

Figure S1 The influence of each study on the association between PLR and prognosis in SCLC patients in different models. (A) the association between PLR and OS in univariate model; (B) the association between PLR and OS in multivariate model; (C) the association between PLR and PFS in univariate model; (D) the association between PLR and PFS in multivariate model.

Table S1 Publication bias - Egger's and Begg's test

	OS-Uni	OS-Multi	PFS-Uni	PFS-Multi
Begg	0.94	0.94	1	0.452
Egger	0	0.417	0.891	0.196

Abbreviations: OS: Overall Survival; PFS: Progression Free Survival; Uni: Univariate model; Multi: Multivariate model

Search strategy

PubMed

#1 (small cell lung cancer) OR (sclc)

#2 ((((overall survival) OR (os)) OR (progression free survival)) OR (pfs)) OR (prognosis)

#3 ((platelet lymphocyte ratio) OR (platelet to lymphocyte ratio)) OR (plr)

#1 AND #2 AND #3

Embase

#1. 'small cell lung cancer'/exp OR 'small cell lung cancer'

#2. Sclc

#3. 'platelet lymphocyte ratio'/exp OR 'platelet lymphocyte ratio'

#4. 'prognosis'/exp OR prognosis

#5. 'progression free survival'/exp OR 'progression free survival'

#6. 'overall survival'/exp OR 'overall survival'

#7. #1 OR #2

#8. #4 OR #5 OR #6

#9. #3 AND #7 AND #8
